# Genetic variations of DNA bindings of FOXA1 and co-factors in breast cancer susceptibility

**DOI:** 10.1038/s41467-021-25670-9

**Published:** 2021-09-13

**Authors:** Wanqing Wen, Zhishan Chen, Jiandong Bao, Quan Long, Xiao-ou Shu, Wei Zheng, Xingyi Guo

**Affiliations:** 1 Division of Epidemiology, Department of Medicine, Vanderbilt University Medical Center, Nashville, TN USA; 2 College of Life Sciences, Fujian Agriculture and Forestry University, Fuzhou, Fujian China; 3 Department of Biochemistry and Molecular Biology & Medical Genetics, & Department of Mathematics and Statistics (Adjunct) Member, Alberta Children’s Hospital Research Institute, Hotchkiss Brain Institute, O’Brien Institute for Public Health, Calgary, AB Canada; 4 Department of Biomedical Informatics, Vanderbilt Epidemiology Center, Vanderbilt-Ingram Cancer Center, Vanderbilt University Medical Center, Nashville, TN USA

**Keywords:** Breast cancer, Cancer epigenetics

## Abstract

Identifying transcription factors (TFs) whose DNA bindings are altered by genetic variants that regulate susceptibility genes is imperative to understand transcriptional dysregulation in disease etiology. Here, we develop a statistical framework to analyze extensive ChIP-seq and GWAS data and identify 22 breast cancer risk-associated TFs. We find that, by analyzing genetic variations of TF-DNA bindings, the interaction of FOXA1 with co-factors such as ESR1 and E2F1, and the interaction of TFs with chromatin features (i.e., enhancers) play a key role in breast cancer susceptibility. Using genetic variants occupied by the 22 TFs, transcriptome-wide association analyses identify 52 previously unreported breast cancer susceptibility genes, including seven with evidence of essentiality from functional screens in breast relevant cell lines. We show that FOXA1 and co-factors form a core TF-transcriptional network regulating the susceptibility genes. Our findings provide additional insights into genetic variations of TF-DNA bindings (particularly for FOXA1) underlying breast cancer susceptibility.

## Abstract (web summary)

The identification of transcription factors (TFs) whose binding sites are affected by risk genetic variants remains crucial. Here, the authors develop a statistical framework to analyse ChIP-seq and GWAS data, identify 22 breast cancer risk-associated TFs and a core TF-transcriptional network for FOXA1 and co-factors.

## Introduction

Identifying transcription factors (TFs) whose DNA bindings are altered by genetic variants that regulate susceptibility genes is imperative for understanding the mechanism of transcriptional dysregulation in disease etiology. Genetic fine-mapping studies in breast cancer suggest that *cis*-regulatory risk variants may disrupt DNA binding affinities of TFs, particularly for known master regulators FOXA1 and ESR1, altering the regulation of gene expression and affecting breast cancer risk^1–7^. A previous study analyzed chromatin immunoprecipitation followed by high throughput sequencing (ChIP-seq) data for TFs, including FOXA1 and ESR1, in breast cancer cell lines to investigate the enrichment of TF-DNA bindings of genome-wide association studies (GWAS)-identified single nucleotide polymorphisms (SNPs)^8^. It found that breast cancer risk-associated regulatory SNPs modulated the binding affinity of FOXA1 and altered gene expression. Two previous integrative data analyses using gene expressions, TF ChIP-seq data, and GWAS-identified SNPs also revealed that breast cancer risk was related to TFs such as ESR1, MYC, KLF4^9^, and others^10^, suggesting the functional role of cancer risk-associated SNPs. Other studies have identified disease-related regulatory elements using epigenetic data such as histone modifications^11–15^. However, previous studies had the suboptimal statistical power to identify disease-associated TFs or elements because they focused on a limited number of GWAS-identified SNPs. Most recently, a statistical approach, GARFIELD^16^, has been developed to identify disease-relevant genomic elements using epigenetic data from the Encyclopedia of DNA Elements (ENCODE) and Roadmap Epigenomics projects and GWAS-identified variants. In the GARFIELD approach, a greedy pruning procedure was proposed to extract a set of independent variants to classify disease-relevant genomic features through the integration of functional annotations with association signals. This approach was conservative due to the potential loss of true causal variants resulting from the greedy pruning, and its statistical power is decreased due to the dichotomization of outcome variables (i.e., GWAS *P*-values). In this work, we develop a computational epigenetic and statistical framework to analyze extensive TF ChIP-seq data (Supplementary Data 1) and GWAS summary statistics data (*n* = 11,337,849 genetic variants) from the Breast Cancer Association Consortium (BCAC) with a goal to establish a landscape of genetic variations for TF-DNA bindings of risk associated TFs for breast cancer.

## Results

### Overview of the developed statistical framework

**Fig. 1:**
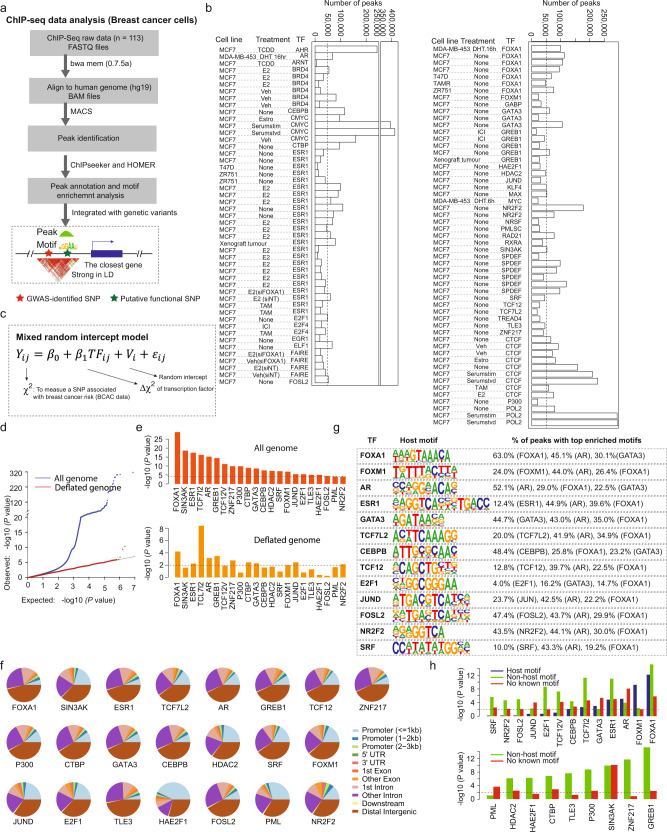
Overview of the developed analytic framework and discovery of risk-associated TFs in breast cancer. **a** A flow chart to illustrate the integrative analysis of ChIP-seq data (*n* = 113) and GWAS summary statistics of genetic variants data (*n* = 11 million) for breast cancer. **b** Barplots showing the numbers of detected binding peaks for each TF ChIP-seq data set in breast cancer cell lines. **c** Generalized mixed models constructed to evaluate the associations between the Chi-squared values (*Y*) and TF-DNA binding status of genetic variants of each TF, given LD blocks of variants to handle their dependence. **d** Quantile–quantile (QQ) plots of the association results of genetic variants in the whole genome (blue) and deflated genome (red) from the BCAC GWAS data. **e** A total of 22 identified TFs with genetic variation of TF-DNA bindings significantly associated with breast cancer risk. Top panel is for the whole genome and the bottom panel is for the deflated genome. **f** Distribution of genomic features (i.e., promoter, enhancer) for peaks of each TF. **g** Significant proportions of the host motifs and other known motifs for 13 TFs were detected based on motif enrichment analysis of their ChIP-seq peaks. **h** Genetic variations of TF-DNA bindings of TFs associated with breast cancer risk in BCAC data, stratified by motifs (i.e., host, non-host, and no motif). Of the 22 identified TFs, 13 with detected host motifs and the remaining ones without detected host motifs were presented in the top and bottom panels, respectively.

To investigate how genetic variations of TF-DNA bindings affect breast cancer susceptibility, we developed an analytic framework to analyze ChIP-seq and breast cancer GWAS summary statistics data (Fig. 1a–c). By analyzing a total of 113 TF ChIP-seq data sets from multiple breast cancer cell lines collected from ENCODE and the Cistrome database (http://cistrome.org/) (Fig. 1a, b, Supplementary Data 1 and “Methods” section), we identified TF-DNA binding regions. An *n* × *m* matrix for *n* = 11,337,849 genetic variants from the BCAC GWAS data was generated with annotation from *m* = 113 TF-DNA binding regions. We used the Chi-squared value for each genetic variant reported in the BCAC GWAS summary data to measure its association with breast cancer risk. We then used generalized mixed models to estimate the associations between the Chi-squared values (*Y*) and TF binding status of genetic variants located in binding sites of each TF given LD blocks of genetic variants to handle the dependence between genetic variants (Fig.1c and Eq. 1). To define approximate independent LD blocks similar to other studies^17,18^, we defined LD blocks using non-overlapping segments of 100 kb (a similar result with 500 kb; see “Methods” section).

1 $${Y}_{{ij}}={\beta }_{0}+{\beta }_{1}{{TF}}_{{ij}}+{V}_{i}+{\varepsilon }_{{ij}}$$

Specifically, ${Y}_{{ij}}$ is the Chi-square value for the *j*th variant in the *i*th LD block; *β*_0_ is the fixed intercept, and *β*_1_ is the fixed slope, which measures the mean difference of the Chi-square values ($\triangle {\bar{\chi }}^{2}$) between TF status; ${{TF}}_{{ij}}$ is the *j*th TF value (i.e., 1 for a variant located in a TF binding site, 0 otherwise) in the *i*th LD block; ${V}_{i}$ is the random intercept for the *i*th LD block; and ${{{{{{\rm{\varepsilon }}}}}}}_{{{{{{\rm{ij}}}}}}}$ is the error term.

### Genetic variations of TF-DNA bindings of breast cancer risk-associated TFs

Using our developed analytic framework, we established a landscape of genetic variations of TF-DNA bindings for 22 breast cancer risk-associated TFs, which were identified at Bonferroni-correction *P* < 0.05 (two-sided). Of them, the top risk-associated TFs included well-known breast cancer master regulators, FOXA1, ESR1, and AR, and other related TFs, such as SIN3AK and TCF7l2 (Fig. 1e and Supplementary Table 1). In addition, we generated a “deflated” genome (Fig. 1d, red line) based on random uniform distribution of GWAS *P*-values after removing variants majorly from those having small *P*-values for breast cancer risk in each block (see “Methods” section). In this “deflated” genome, we still observed that genetic variations of TF-DNA bindings for 17 TFs remained significant at a nominal *P* < 0.05. The findings not only support the associations of these TFs but also imply additional genetic susceptibility is likely conferred by non-GWAS significant genetic variants occupied by these TFs. The associations for the other five TFs (P300, SRF, E2F1, HAE2F1, and FOSL2) were not significant, perhaps due to a decreased number of TF bindings, or they may be sensitive to this conservative approach (Fig. 1e and Supplementary Table 1). Comparing TF frequencies of the genomic background, we confirmed that the TF-DNA binding sites of the identified TFs were significantly enriched in both GWAS-identified variants and their flanking regions (±500kb) (Supplementary Table 1).

### Motif-dependent genetic variations of TF-DNA bindings of breast cancer risk-associated TFs

Genomic annotation of the 22 identified TFs’ binding sites revealed that they are generally significantly enriched in intragenic regions. TFs such as HAE2F1, PML, FOXM1, and JUND showed proximal promoter binding patterns, while other TFs such as FOXA1, ESR1, and GATA3 and the enhancer marker P300 showed distal binding patterns (Fig. 1f). We performed motif enrichment analysis based on the ChIP-seq binding regions for each TF (see “Methods” section). We observed a significantly increased proportion of the host motifs for 13 TFs, with the top five TFs being FOXA1 (63%), AR (52.1%), CEBPB (48.4%), FOSL2 (47.4%), and GATA3 (44.7%) (Fig. 1g). The dominant motif of FOXA1 was particularly enriched in other TFs, suggesting it may interact with other TFs to co-occupy binding sites of *cis*-regulatory elements (Fig. 1g). To further illustrate the effects of genetic variations of TF-DNA bindings in a motif-dependent manner, we analyzed their associations with breast cancer risk stratified by motif status (presence of the host motif, non-host motif, and no known motifs) (see “Methods” section). Overall, we found that the associations of breast cancer risk with TFs were particularly evident with the host or known-enriched motifs (Fig. 1h and Supplementary Table 2).

### Genetic variations of TF-DNA bindings of FOXA1 and co-factors driving breast cancer susceptibility

**Fig. 2:**
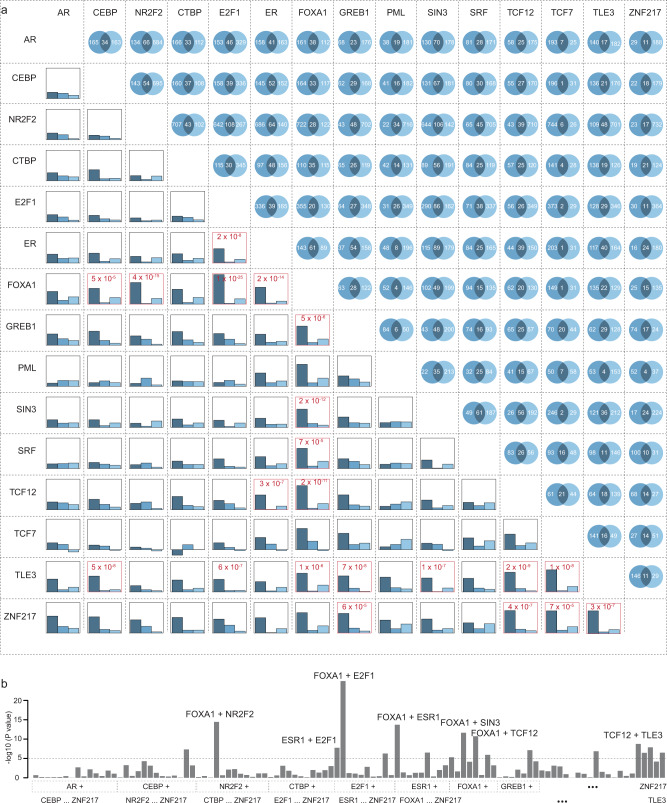
Association of co-occupancy of two TFs with breast cancer risk in BCAC data. **a** In the upper-right triangle, the numbers of genetic variants (multiplied by 1000) that are occupied by specific TFs or co-occupied by two TFs were showed for each TF pair. In the lower-left triangle, barplots showing the association strengths (regression coefficients) for the genetic variants occupied by two TFs (only first TF and only second TF, respectively) as indicated by dark blue to gray-blue colors. Two TFs with significant interactions at *P* < 1 × 10^−^^5^ were highlighted in red. **b** Barplots show interaction between two TFs with significant interactions indicated on the top: FOXA1 + E2F1, FOXA1 + NR2F2, FOXA1 + ESR1, FOXA1 + SIN3, and FOXA1 + TCF12, at *P* < 1 × 10^−5^.

To investigate whether genetic variations of TF-DNA bindings of multiple core TFs conferred breast cancer risk more than a single TF, we first analyzed co-occupied binding regions of the identified 22 risk-associated TFs (see “Methods” section). We observed a substantial proportion of genetic variants located in co-occupied binding sites (Fig. 2a and Supplementary Data 2). Pair-wise analyses among the 22 TFs showed significant interactions at *P* < 0.0002 (0.05/231 possible TF pairs from 22 TFs) among 15 TFs. In general, the associations of breast cancer risk with genetic variants co-occupied by two TFs were significantly stronger than those occupied by a single TF, as compared with genetic variants not occupied by any TFs. The AR-PML pair was an exception, where the associations for genetic variants co-occupied by this TF pair were weaker (Supplementary Data 2). We observed that the top five strongest TF co-occupancy pairs associated with breast cancer risk were FOXA1 + E2F1, FOXA1 + NR2F2, FOXA1 + ESR1, FOXA1 + SIN3, and FOXA1 + TCF12 (Fig. 2a, b and Supplementary Data 2). The interaction effects on breast cancer risk of these five TF pairs were all highly significant (*P* < 1 × 10^−5^; Supplementary Data 2).

**Fig. 3:**
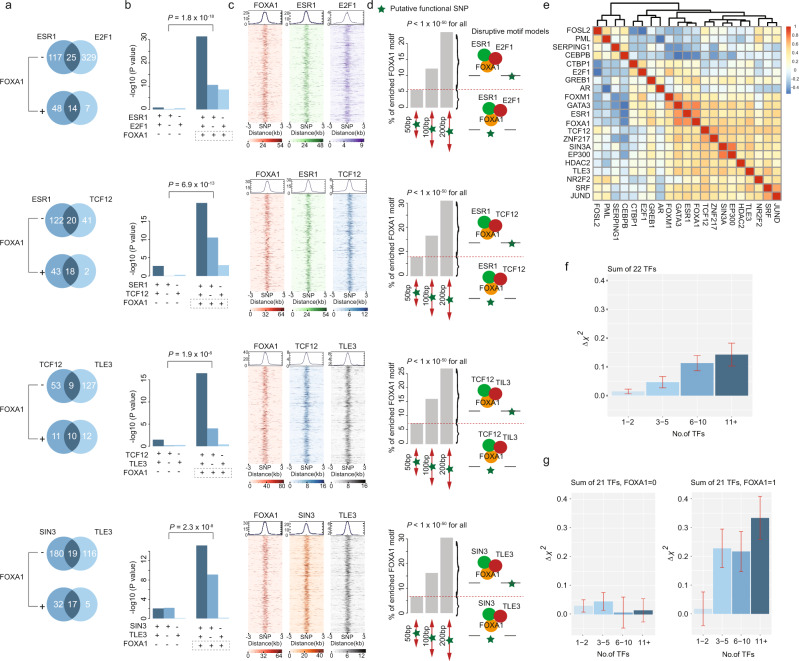
FOXA1 interacting with co-factors, driving breast cancer susceptibility. **a** The numbers of genetic variants (multiplied by 1000) that are occupied by specific TFs or co-occupied by two TFs, stratified by FOXA1. **b** Barplots show the association strengths for genetic variants occupied by specific TFs occupancy. The “+” and “−” symbols underneath the *x* axis represents genetic variants with and without occupancy of specific TFs. *P*-value on the top shows the significance of the interaction between FOXA1 and TF pairs. **c** Heatmap shows the TF binding signals for the flanking regions of genetic variants co-occupied by FOXA1 and TF pairs. The heavy color in each panel denotes the binding signal of the TF of interest. **d** Barplots show the proportion of the detected FOXA1 motif based on motif enrichment analysis for flanking regions of genetic variants (i.e., ±25, 50, and 100 bp). Disruptive motif models are illustrated in the right panel. **e** Heatmap shows a correlation pattern based on gene-expression data of 22 TFs in the GTEx data. The color change from blue to red denotes the correlation coefficients changes from −0.5 to 1. **f** Barplots show association (regression coefficients) of TF score with breast cancer risk in BCAC data. **g** Barplots show an association of TF score with breast cancer risk in BCAC data, stratified by FOXA1. In these figures, the two-sided nominal *P*-values and $\triangle {\chi }^{2}$ were derived from the generalized mixed models and the error bars denote 95% confidence intervals.

We then investigated the associations of breast cancer risk with core TF-DNA bindings of three TFs. FOXA1 showed particularly significant interactions with other TF pairs. The associations of breast cancer risk for ESR1-E2F1, ESR1-TCF12, TCF12-TLE3, and SIN3-TLE3 pairs were significantly stronger in loci also occupied by FOXA1 (*P* < 1 ×10^−5^; Fig. 3a, b and Supplementary Table 3). The genetic variants co-occupied by three of these TFs were clustered in the peak center within each of the co-occupied TFs, supporting they may play disruptive roles in the TF-DNA binding (Fig. 3c). To examine whether these genetic variants may alter TF-DNA binding affinities via a motif-dependent mechanism, we further performed motif enrichment analysis within fragments of 50, 100, and 200 bp (centered with genetic variants) for the regions co-occupied by FOXA1 and two co-factors. We observed that 5–10% of the most over-represented FOXA1 motifs (10 bps) were presented in these fragments of 50 bp, indicating the genetic variants within motifs may directly lead to the disruption of these motifs (motif-dependent model) (Fig. 3d, *P* < 1 × 10^−50^ for all). We observed a 15–30% proportion of the FOXA1 motif in the fragments of 100 bp and 200 bp (two or three-fold enrichment compared to the fragments of 50 bp), implying the motif-independent model of these genetic variants may also play a significant role in affecting TF-DNA binding affinities (Fig. 3d, *P* < 1 × 10^−50^ for all). These findings provide evidence that genetic variations of TF-DNA binding from the pioneer factor FOXA1 and co-factors may control the core transcriptional regulatory circuitry, and confer breast cancer susceptibility.

We further analyzed the correlations of TFs based on gene-expression profiles in normal breast tissue from Genotype-Tissue Expression (GTEx) data. We observed a large number of highly correlated TFs (Fig. 3e). To evaluate the association of breast cancer risk with genetic variations of multiple TFs, we defined a TF score as the total number of TF-DNA bindings of the 22 identified TFs (Fig. 3f, Supplementary Table 4, and see “Methods” section). Our results showed that higher TF scores were associated with higher breast cancer risk with a linear trend. Stratified by FOXA1, the linear associations between breast cancer risk and TF scores of the other 21 TFs were significantly stronger in genetic variants occupied by FOXA1 than those not occupied by FOXA1 (*P* for interaction = 1.2 × 10^−18^; Fig. 3g and Supplementary Table 4).

### Genetic variations of TF colocalizing with chromatin features associated with breast cancer risk

**Fig. 4:**
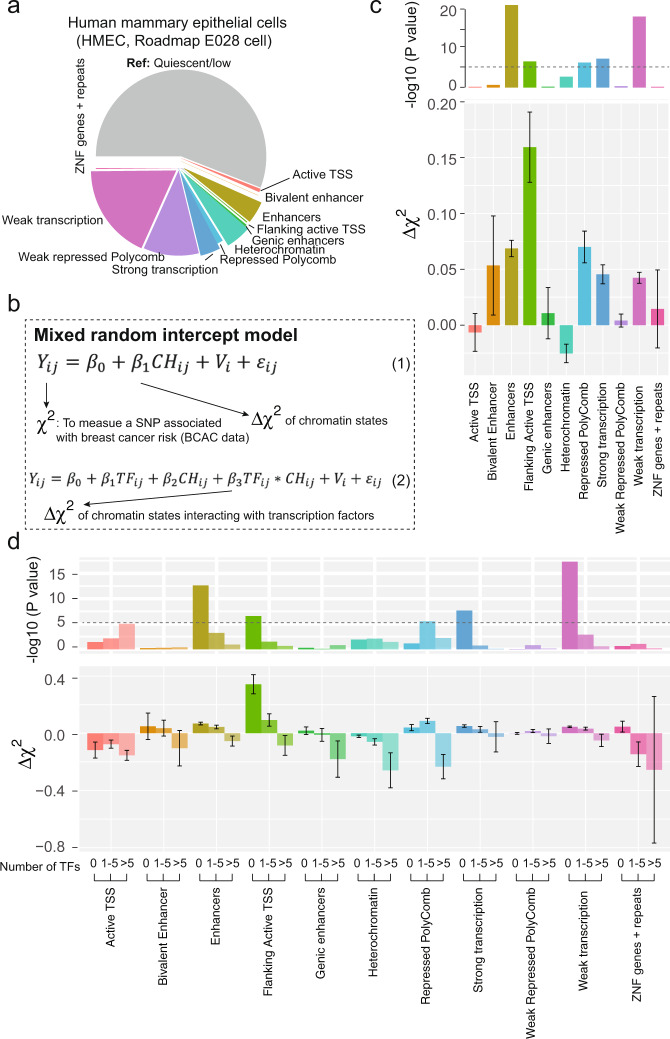
TFs colocalizing with chromatin features associated with breast cancer risk. **a** Distribution of chromatin features annotated from chromatin states using the ChromHMM tool in breast human mammary epithelial cell (HMEC). Different colors denotes different chromatin states. **b** Mixed random intercept model constructed to evaluate associations of breast cancer risk with chromatin states using formula (1) and its interaction with TF-occupancy score using formula (2). **c** Barplots show the association of breast cancer risk with each chromatin feature (upper panel for *P*-values and lower panel for regression coefficients and standard errors). **d** Barplots show association with breast cancer risk for each chromatin feature (upper panel for *P*-values and lower panel for regression coefficients and standard errors), stratified by TF scores. In these figures, the two-sided nominal *P*-values and $\triangle {\chi }^{2}$ were derived from the generalized mixed models and the error bars denote 95% confidence intervals.

We evaluated the associations of breast cancer risk with chromatin features (defined as chromatin states annotated from ChromHMM^19^) in human mammary epithelial cells (HMEC, Roadmap E027) and myoepithelial primary cells (Roadmap E028) (see “Methods” section). Compared with quiescent/low chromatin features, we consistently observed in both cell lines that genetic variants located in enhancers, flanking active transcription start sites (TSS), and strong or weak transcription sites were associated with significantly higher breast cancer risk, while genetic variants located in heterochromatin were associated with significantly lower breast cancer risk (Fig. 4a–c and Supplementary Data 3). To further evaluate whether the effects of TF occupancies are influenced by colocalization of chromatin features, we analyzed the interactions of chromatin features and TF scores (categorized as 0 TF, 1–5 TFs, and 6–22 TFs) on breast cancer risk. We found that higher breast cancer risk associated with enhancers and strong/weak transcriptions were mainly in loci with low TF scores, while lower breast cancer risk associated with heterochromatin were mainly in loci with high TF scores, with significant interactions (*P* = 6 × 10^−5^; Fig. 4d; Supplementary Data 3).

### Discovery of putative susceptibility genes with TWAS analysis

**Fig. 5:**
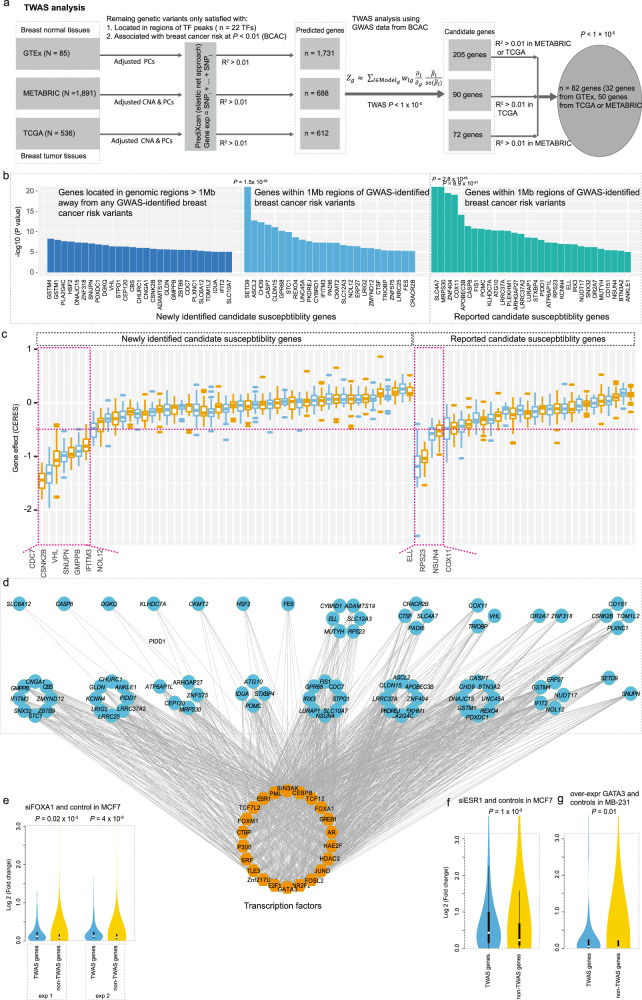
TWAS analysis using an improved model building and core TF-transcriptional network regulating the identified susceptibility genes formed by FOXA1 and co-factors. **a** Flow chart to illustrate the TWAS analysis using the improved model building based on putative regulatory variants occupied by the identified 22 risk-associated TFs. **b** Barplots show association of breast cancer risk with TWAS-identified genes, separated by previously unidentified ones: far away GWAS loci (>1 Mb) and within GWAS loci (<1 Mb), and previously reported ones. **c** Boxplot shows TWAS-identified genes effects on cell proliferation using experimental data from CRISPR (Avana) public 20Q3. A total of 11 genes, including seven previously unidentified (left panel) and four previously reported ones (right panel) showed evidence of essentiality on cell proliferation based on a cutoff of median CERES values < −0.5. **d** The TWAS-identified genes regulated by TF networks based on the putative regulatory variants occupied by the identified TFs. **e**–**g** Boxplots show that TWAS-identified genes had higher folds of changes than non-TWAS-identified genes, based on gene-expression data generated by FOXA1 (**e**), ESR1 (**f**), and GATA3 (**g**) silence/over-expression and control breast cancer cells. In the boxplots shown in these figures, the whiskers denote the range, the boxes denote the interquartile range; the middle bars in **c** or middle white points in **e**–**g** denote the median, and the violin shapes in **e**–**g** represent the data distribution. The two-sided nominal *P*-values shown in **e**–**g** were derived from the Wilcoxon-rank sum test.

We built gene-expression prediction models using only putative regulatory genetic variants (*n* = 68,039) located in the binding sites of the 22 identified risk-associated TFs with reported *P* < 0.01 by the BCAC GWAS data (see “Methods” section). Even though we only used these putative regulatory genetic variants, we were able to predict gene expressions at *R*^2^ > 0.01 for 7538 genes using data from GTEx, which is only slightly less than the total number (*n* = 9109) of predicted genes using all genetic variants in previous breast cancer TWAS^20^. We further focused solely on genes that can be predicted by the same set of local genetic variants from either of The Cancer Genome Atlas (TCGA) or the Molecular Taxonomy of Breast Cancer International Consortium (METABRIC) at *R*^2^ > 0.01 (Fig. 5a; see “Methods” section). By applying the models to BCAC GWAS data, we identified 82 genes with predicted expressions that were associated with breast cancer risk at *P* < 1 × 10^−5^, with 73 genes reaching *P* < 5 × 10^−6^ at a Bonferroni-corrected significance level, as applied in previous breast cancer TWAS in which 48 genes was identified using regular TWAS. Specifically, we identified 27 significant genes located in regions not yet identified by GWAS^21^ (1 Mb away; Fig. 5b; Supplementary Data 4). In addition, we uncovered 25 significant putative breast cancer risk genes in known GWAS loci that had not been previously reported (Fig. 5b and Supplementary Data 4).

We further explored the functional roles of the 82 TWAS-identified genes using CRISPR silencing data from gene essentiality screens in 34 breast-relevant cell lines (see “Methods” section)^22^. Using similar cutoffs of median CERES Score < −0.5 in the above cells, following previous literature^22,23^, we discovered seven previously unreported genes (*CDC7*, *CSNK2B*, *VHL*, *SNUPN*, *IFITM3*, *GMPPB,* and *NOL12*) and four previously reported genes (*ELL*, *RPS23*, *NSUN4*, and *COX11)*, which showed evidence of essentiality on cell proliferation (Fig. 5c). Overall, we observed that a total of 46 genes (56%) showed a trend of essentiality on cell proliferation, at *P* < 6.2 × 10^−4^, a Bonferroni-corrected significance level. Notably, many of these previously unidentified breast cancer susceptibility genes, such as *GSTM4*^24^, *GSTM1*^25^, *HSF2*^26^, *DNAJC15*^27^, *SNUPN*^28^, *DGKQ*^29^, *VHL*^30^, *CHURC1*^31^, *CBS*^32^, *CSNK2B*^33^, *CDC7*^34^, *IDUA*^35^, and *IFIT2*^36^ are involved in cancer biology.

### FOXA1 and co-factors form a core TF-transcriptional network regulating breast cancer susceptibility genes

Using TWAS-identified genes, we investigated the TF-DNA bindings of genetic variants that were predictors for the expressions of each gene. We observed that these putative susceptibility genes were co-regulated by multiple TFs. Specifically, most of these genes (78 out of 82) were shown to be regulated by at least five TFs, with the exception of *SLC6A12*, *CASP8*, *DGKQ,* and *KLHDC7A* (Fig. 5d). Of note, we observed that the three previously reported with experimental verification (*SSBP4*, *MRPS30,* and *ATG10*) were all co-occupied by at least 10 TFs^37^. Of the identified 22 TFs, 18 were observed to likely regulate at least 50% of these putative susceptibility genes, except for the TFs PML (49%), TCF7L2 (41%), ZNF217 (28%), and histone acetyltransferase P300 (38%). Of particular interest, these putative susceptibility genes were mostly regulated by three known master regulators: FOXA1 (61%), GATA3 (80%), and ESR1 (74%). To further verify that these genes were regulated by these three TFs, we analyzed gene-expression data from knockdown (FOXA1 and ESR1) and over-expression (GATA3) experiments in breast cancer cell lines (see “Methods” section). Our results showed that TWAS-identified genes were significantly differentially expressed compared with the background genes, supporting that they were regulated by FOXA1, ESR1, and GATA3 (Fig. 5e–g).

## Discussion

Genetic studies over the past decades, including our own studies, have identified multiple candidate susceptibility genes for GWAS-identified risk loci through the integrative analyses of eQTL and GWAS data^9,37–44^. However, the underlying regulatory mechanisms involving specific TFs and functional genetic variants for identified susceptibility genes remained unclear. Our established landscape of TF-DNA bindings of risk-associated TFs and TF-based regulatory elements (i.e., together with chromatin features) provide additional insights into TF-mediated gene regulation for breast cancer genetic susceptibility. In particular, the integration analysis of those putative regulator genetic variants occupied by risk-associated TFs with gene-expression data may improve the discovery of causative genes, with evidential support by the potential regulatory mechanisms. Thus, our approaches and findings may help overcome the challenges of pleiotropy and linkage scenarios of current statistical approaches for susceptibility gene discovery^44,45^. In regular TWAS approaches, the prediction accuracy of the prediction model with *cis*-genetic variants could be low or compromised if they occur in non-regulatory elements (i.e., not in LD with regulatory variants), or if they disrupt binding sites of non-transcribed TFs in target tissues. In addition, LD among genetic variants used in the gene prediction models induces significant gene-trait associations at nearby non-causal genes in the region, leading to false-positive errors. We demonstrated that TWAS analysis using genetic variants located in binding sites of risk-associated TFs significantly improved the detection of breast cancer susceptibility genes. Of note, a total of 1815 unique genetic variants were included in the gene-expression prediction models for the 82 TWAS-identified genes. Of them, 1345 (74%) were annotated within either promoter or enhancer activity regions (Supplementary Data 5; see “Methods” section). We found that 58 (71%) were the closest genes for at least one genetic variant included in a prediction model, including 42 genes that are most likely proximally regulated by putative functional genetic variants with promoter activities (Supplementary Data 5). We found an additional 16 genes that showed evidence of distal regulations by putative functional genetic variants via promoter-enhancer interactions (Supplementary Data 5). Taken together, these 74 genes (90%) showed evidence of regulations by putative functional genetic variants via proximal promoter or distal enhancer–promoter interactions, suggesting they were identified target genes for putative regulatory genetic variants.

We compared our approach with the existing approach for partitioning heritability developed by Finucane and colleagues^14^. We conducted stratified LD score regression to analyze partitioning heritability of TF-based functional categories of the genome. We selected 40 unique TFs from the 113 TF Chip-seq data sets to define the functional categories, including 22 of the identified breast cancer risk-related TFs. The results from the LD score regression are generally consistent with the results with the generalized mixed models (Supplementary Fig. 1). Of the 22 identified TFs, we observed that 17 TFs reached *P* < 1 × 10^−4^, the significance cutoff. However, the other five TFs failed to reach the significance level. On the other hand, among the 18 non-significant TFs in our analysis using mixed models, two TFs (MAX and RXRA) reached the significance level at *P* < 1 × 10^−4^. By comparison, the results from the mixed models are generally more significant than those from the LD score regression. The most important TFs for breast cancer, such as FOXA1 and ESR1, are among the most significant results from the mixed model but less evident in the results from the LD score regression.

It is generally believed that genetic variants located in enhancers and promoters have stronger associations with breast cancer risk than those located elsewhere, which is supported by our findings using data from chromatin states (Fig. 3 and Supplementary Data 3). In addition, we observed significant interactions between chromatin features and TF scores, with higher TF scores of variants located in enhancers and promoters associated with lower breast cancer risk. These findings provide additional insight into understanding the interplay between TFs and *cis*-regulatory elements, which play diverse roles in contributing to the risk of breast cancers.

A limitation of this study is that while the summary statistics of GWAS used in our study were derived from the study participants from all breast cancer subtypes, TF ChIP-seq data were primarily from cell lines of ER^+^ breast cancer (e.g., MCF7) and ChIP-seq data from cell lines of ER^−^ breast cancer were limited. However, this limitation would not affect our main results and conclusions, because (i) ER^+^ cases constitute the majority (about 80%) of breast cancer and (ii) although binding sites of TFs may slightly vary between ER^+^ and ER^−^ subtype, we found that the association of breast cancer risk with genetic variations of TF-bindings remained significant for most of our reported TFs (77%) even after we used the aggressive pruning strategy to remove all of the enriched variants which were significantly associated with breast cancer risk (i.e., deflated genome; Supplementary Table 1). We further analyzed a subset of genetic variants located in TF-DNA binding regions with a detected host or known breast cancer risk-related TF motifs, and we still observed significant associations of breast cancer risk with the identified TFs (Fig. 1h). Our findings on the association of breast cancer risk with the identified TFs are robust. Future studies to analyze epigenome profiles and GWAS data in specific breast cancer subtypes are warranted to identify and differentiate TFs for the risk of specific subtypes of breast cancer.

In summary, our study established the landscape of genetic variations for TF-DNA bindings in association with breast cancer risk by identifying 22 breast cancer risk-associated TFs. Genetic variations occupied by risk-associated TFs are valuable for future fine-mapping of disease-associated variants and TWAS studies. Our approaches can be applied to other human cancers and chronic diseases which have comprehensive ChIP-seq and large-scale GWAS data. Our approaches and findings can help advance the general understanding of genetic and molecular mechanisms underlying human disease and cancer phenotypes.

## Methods

### Data sets

Summary statistics of GWAS data for breast cancer were downloaded from the BCAC. The BCAC is an international, multidisciplinary consortium designed to identify genetic susceptibility factors that are related to the risk of breast cancer. The BCAC have generated GWAS data for a total of 122,977 cases and 105,974 controls from European descendants.

For TCGA data, we used RNA-seq and copy number alteration data downloaded from cBioPortal. We used genetic variants data genotyped by the Affymetrix SNP 6.0 from TCGA’s data portal. Genotype data together with matched gene expressions, somatic copy number alterations in 536 tumor tissue samples from the TCGA were included in this analysis. In the GTEx release 6, there are 85 breast normal tissue samples that were profiled by RNA-seq and the Illumina OMNI 2.5M or 5M SNP Array. We downloaded both genotype and gene-expression data from these samples. Genotype data were processed according to the GTEx protocol. In brief, we excluded variants with a call rate <98%, with differential missingness between the two array experiments (5 M/2.5 M Arrays), with Hardy–Weinberg equilibrium *P*-value < 10^−6^ (among subjects of European ancestry) or showing batch effects. The genotype data were imputed to the Haplotype Reference Consortium reference panel using Minimac3 for imputation. We only used variants with high imputation quality (*R*^2^ ≥ 0.8), minor allele frequency ≥ 0.05, and those included in the HapMap Phase 2 version for expression prediction model building. For data from the METABRIC, we downloaded normalized gene expression and somatic copy alteration data from the cBioPortal. Genetic variant data, genotyped using array-based Affymetrix SNP 6.0 in a total of 1992 samples, were downloaded from EBI (EGAD00010000164). A total of 1891 tumor tissue samples with matched gene expressions, somatic copy number alterations, and genetic variants data from the METABRIC were included in this analysis.

We systematically searched ChIP-seq data of TFs generated in breast cancer cell lines from ENCODE, the Cistrome database, Gene Expression Omnibus (GEO), and literature (Supplementary Data 1). After evaluating their quality control (QC) in previous publications, we collected 113 ChIP-seq data sets (corresponding to 40 TFs) with high qualities for our downstream analyses.

### ChIP-seq data analysis

ChIP-seq data generated from previous studies were listed in Supplementary Data 1. The raw sequencing reads from TF and matched input DNA (if available) were mapped to the human reference genome (hg19) using the Burrows–Wheeler Aligner (BWA) meme program (version 0.7.9a)^46^. The mapped BAM files were further used for the downstream peak calling and density signal visuals using the Integrative Genomics Viewer (IGV, version 2.9) tool. We applied the MACS tool (version 1.4) to identify binding regions (i.e., peaks) of each TF. Binding regions were identified using a stringent criterion at a score >30. We further evaluated the global binding occupancy for each TF in the human genome using the tool ChIPseeker^47^. Specifically, we calculated the frequencies of the identified peaks in the proximal promoter (≤1 kb of TSS), Promoter (1–2 kb of TSS), Promoter (2–3 kb of TSS), 5′ UTR, 3′ UTR, 1st Exon, Other Exon, 1st Intron, Other Intron, Downstream, and Distal Intergenic regions.

### Motif enrichment analysis

We used HOMER software^48^ for motif analysis based on the peak files (“summits.bed” file) generated from the ChIP-seq data analysis by MACS. We extracted DNA sequences of 250 bp regions from the center of each peak. The findMotifsGenome.pl script in HOMER was used to discover motifs for each peak. The enrichment of a motif in TF peaks was then calculated as the ratio of the motif occurrence frequency in TF peaks to its corresponding frequency in background sequences and the significance was calculated based on the binomial distribution. Details in enrichment analysis have been described (http://homer.ucsd.edu/homer/motif/index.html). In our analysis, we reported known motifs based on the motif collections including JASPAR which was described on the HOMER website (http://homer.ucsd.edu/homer/motif/motifDatabase.html). Similarly, we also conducted motif enrichment analysis for flanking regions on genetic variants of interests (i.e., ±25, ±50, ±100, and ±250 bp). The percentage and significance of known enriched motifs were reported for each set of fragments.

### Generalized mixed models

We used generalized mixed models to account for variant correlation within LD blocks. Because Chi-square values are strictly positive, generalized mixed models with errors from a gamma distribution should be used. In this study with such a large sample size, generalized mixed models with a Gaussian error term are also appropriate. We used both approaches in the analyses and found similar results. We reported the results with the latter approach for easier interpretation. We defined LD blocks using non-overlapping segments of 100 kb (a similar result with 500 kb). We used generalized mixed models given LD blocks to investigate genetic variations of TF-DNA bindings associated with cancer risk, which was measured with both continuous Chi-square values reported in GWAS data and binary GWAS p-values cut at a certain threshold (e.g., *P* < 5 × 10^−8^).

BCAC GWAS summary data were highly enriched with genetic variants with large Chi-square and small *P*-values. We generated a “deflated” genome based on a random uniform distribution of GWAS *P*-values, which removed many genetic variants with small *P*-values for breast cancer risk in each block. In this way, we were able to evaluate the enrichment of TFs in potential breast cancer risk loci with a conservative approach.

To investigate associations of breast cancer risk with variations of TF-DNA binding by a single TF (Eq. 1) or multiple TFs (Eq. 2), we used the above-proposed generalized mixed model approach. The generalized mixed models have the forms:

2 $${Y}_{{ij}}={\beta }_{0}+{\beta }_{1}{{TF}1}_{{ij}}+{\beta }_{2}{{TF}2}_{{ij}}+{\beta }_{3}{{TF}1}_{{ij}}\times {{TF}2}_{{ij}}+{V}_{i}+{\varepsilon }_{{ij}}$$

In Eq. 2, ${Y}_{{ij}}$ is the Chi-square value for *j*th variant in *i*th LD block; ${\beta }_{0}$ is the fixed intercept, ${\beta }_{1}$, and ${\beta }_{2}$ are the fixed slopes for the main effect of ${{TF}}_{{ij}}$, ${\beta }_{3}$ is the fixed effect of the interaction term for two TFs, ${V}_{i}$ is the random intercept for *i*th LD-block, and ${\varepsilon }_{{ij}}$ is a Gaussian error term. The interactions of TFs and chromatin features were also evaluated using a similar approach.

We analyzed all 40 TFs from the 113 ChIP-seq data sets generated in breast cancer cell lines to evaluate their associations with breast cancer risk. We identified 22 significant TFs with *P* ≤ 1 × 10^−4^, which reached the Bonferroni-correction threshold even though the number of independent tests was 113 (0.05/113 = 4.4e−4). The smallest *P*-value among other TFs was 3.7e−3, which did not reach the Bonferroni-correction threshold even though the number of independent tests was only 14. Based on this consideration, we decided to use the stringent *P* ≤ 1 × 10^−4^ to define the significant TFs. We observed comparable association significances for the same TF in multiple cell lines, as a majority of TF occupancies overlap across these cells. We chose the cell type with the most significant association and used the data from the cell-type-specific TF occupancy for our downstream analyses. Based on these analyses, we were able to evaluate the effects of co-occupancy of TFs and interactions of TF-chromatin features on breast cancer risk and provided a landscape of genome-wide variations of cancer-relevant TF-DNA bindings.

### Gene-expression prediction model building

Genetic and transcriptome data from breast normal tissue samples from GTEx, and breast cancer tumor tissue samples from TCGA and METABRIC were used to build gene-expression prediction models in this study. Data processing for all data sets was described in our previous study^37^. In brief, for genotype data, the genetic variants data were imputed using the reference genome from the 1000 Genomes project with the Minimac tool^49^, implemented in the Michigan Imputation server. Only common genetic variants (minor allele frequency > 0.05) with high imputation quality (*R*^2^ > 0.3) were included. Genetic variants with a call rate < 98%, with a Hardy–Weinberg equilibrium *P* < 10^−6^ or showing batch differences were excluded. Principal component analysis (PCA) was conducted using EIGENSTRAT^50^ to generate top PCs from the genotype data. For gene-expression data, expression levels of each gene were measured using reads per kilobase per million (RPKM). We performed data QC and normalization processing by filtering low-expressed genes, log2 transforming, and Robust Multichip Average (RMA). We further performed rank-based inverse normal transformation for gene-expression levels across all samples. We performed a probabilistic estimation of expression residuals (PEER) analysis to adjust for batch differences and other potential confounding factors^51^ for downstream prediction model building. Expression levels of pseudogenes were not included in our analysis because of concerns for potential inaccurate measurements^52^.

We trained the gene-expression prediction model by flanking genetic variants (flanking ±1 Mb region) using an elastic-net approach. For each gene, the gene-expression level was regressed on the number of effect alleles (0–2) for each genetic variant with adjustment for top PCs, sex, age, potential batch effects, and other potential confounding factors (PEERs). For data from breast cancer tumor tissues in TCGA and METABRIC, we have additionally adjusted CNA in the models. We only used approximately 68k genetic variants with reported GWAS *P* < 0.01 and that were occupied by any of the 22 identified TFs. Prediction model performance was assessed using 10-fold cross-validation and the explained variance (*R*^2^).

### Association analyses between predicted gene expression and breast cancer risk

To identify susceptibility genes for breast cancer risk, we applied the weight matrix obtained from the gene prediction models to the summary statistics from the BCAC GWAS data set using the MetaXcan tool^53^. The MetaXcan method, described elsewhere^20,54^, was used for association analyses.

3 $${Z}_{g}\approx \mathop{\sum }\limits_{l\in {{{{{{\rm{Model}}}}}}}_{g}}{w}_{{lg}}\frac{{\hat{\sigma }}_{l}}{{\hat{\sigma }}_{g}}\,\frac{{\hat{\beta }}_{l}}{{{{{{{\rm{se}}}}}}}(\hat{\beta }_{l})}$$

In Eq. 3, the *Z*-score was used to estimate the association between predicted gene expression and breast cancer risk. Here, ${w}_{{lg}}$ is the weight of genetic variant $l$ for predicting the expression of gene $g$. ${\hat{\beta }}_{l}$ and ${{{{{{\rm{se}}}}}}}({\hat{\beta}}_{l})$ are the GWAS-reported regression coefficients, and its standard error for variant $l$, and ${\hat{\sigma }}_{l}$ and $\,{\hat{\sigma }}_{g}$ are the estimated variances of variant $l$ and the predicted expression of gene $g,$ respectively.

We conducted the association analyses separately using gene-expression data from GTEx, TCGA, or METABRIC. The genes significantly associated with breast cancer risk were identified based on the criteria: *R*^2^ > 0.01 in two of these three gene-expression data sets and minimum *P*-values from the three data sets <1 × 10^−5^.

### ChromHMM annotation and chromatin–chromatin interaction data analysis

Functional annotation was evaluated using epigenetic data from both ENCODE and Roadmap projects. For each genetic variant, we investigated whether variants were mapped to functional regions (i.e., promoter or enhancer) using chromatin states annotation in HMEC (Roadmap E028 cell) and myoepithelial primary cells (Roadmap E027 cell) and the database HaploReg v4^55^. In addition, experimentally derived chromatin interactions generated by Hi-C, ChIA-PET, and IM-PET were collected from the 4DGenome database^56^. Additional chromatin interactions data from breast cancer cells were also analyzed^57^. To further analyze chromatin–chromatin interactions between the regions for functional genetic variants and promoter regions of the identified candidate susceptibility genes, we examined whether functional genetic variants were mapped to ±2 kb flanking regions of the TSS to determine their chromatin–chromatin interactions.

### Effect of gene silencing on cell proliferation using data from CRISPR–Cas9 essentiality screens in breast relevant cells

To investigate the effect of an individual gene on essentiality for proliferation and survival of cancer cells, we downloaded two comprehensive data sets including “sample_info.csv” and “Achilles_gene_effect.csv” from the DepMap portal (https://depmap.org/portal/). These data provided estimated gene-dependency levels from CRISPR–Cas9 essentiality screens for a total 18,119 genes using a computational method, CERES^22^. For each gene, we tested its significance on cell proliferation based on the count of negative CERES values in a total of 34 breast-relevant cells using the Binomial test. The median CERES value of the 34 breast-relevant cells was also calculated for each gene. The cutoff of CERES value = −0.5 was used to show a gene’s evidence of essentiality^22,23^.

### Knockdown experiment data for the TF FOXA1, ESR1, and GATA3

To investigate genes regulated by TFs FOXA1, ESR1, and GATA3, we analyzed gene-expression data from TF knockdown (FOXA1 and ESR1) and over-expression (GATA3) experiments in breast cancer cell lines from previous literature. Gene-expression data from FOXA1 knockdown experiments in breast cancer MCF7 cells were downloaded from NCBI using accession number GSE25315, which included two small-interfering (si) RNAs to silence FOXA1 with three replicates: (i) si-FOXA1 vs si-Control and vehicle, and (ii) si-FoxA1 and si-Control and ESR1. Gene-expression data from small hairpin (shRNA) plasmid transfection to silence ESR1 in MCF7 and over-expression for GATA3 in MDA-MB-231 cell lines with three replicates for each were downloaded from NCBI using accession numbers GSE27473 and GSE24249, respectively. We analyzed the normalized gene-expression data and calculated the fold change of each gene using the mean values of biological replicates between silence/over-expression treated cells and control cells. To evaluate whether TWAS-identified genes were distinct from transcriptome background genes, Wilcoxon signed-rank test was used to compare the fold change of expression between these two gene subsets.

### Reporting summary

Further information on research design is available in the Nature Research Reporting Summary linked to this article.

## Supplementary information

Supplementary Information

Description of Additional Supplementary Files

Supplementary Data 1-5

Reporting Summary

## Data Availability

Summary statistics of GWAS data for breast cancer were downloaded from the BCAC website (http://apps.ccge.medschl.cam.ac.uk/consortia/bcac/). ChIP-seq data in breast cancer cell lines were collected from the ENCODE (https://www.encodeproject.org/) and the Cistrome database (http://cistrome.org/) (accession numbers described in Supplementary Data 1). Chromatin states annotation in HMEC (Roadmap E028 cell) and myoepithelial primary cells (Roadmap E027 cell) can be accessed from Roadmap Project (http://www.roadmapepigenomics.org/). Gene expression and genotype data in breast cancer were collected from the GTEx (https://gtexportal.org/home/), TCGA (https://portal.gdc.cancer.gov/), cBioPortal (https://www.cbioportal.org/), and the METABRIC (https://ega-archive.org/studies/EGAS00000000083). To investigate the effect of an individual gene on essentiality for proliferation and survival of cancer cells, we collected two comprehensive data sets including “sample_info.csv” and “Achilles_gene_effect.csv” from the DepMap portal (https://depmap.org/portal/). Gene-expression data from FOXA1 knockdown experiments in breast cancer MCF7 cells were downloaded from NCBI using accession number GSE25315. Gene-expression data from shRNA plasmid transfection to silence ESR1 in MCF7 and over-expression for GATA3 in MDA-MB-231 cell lines were downloaded from NCBI using accession numbers GSE27473 and GSE24249, respectively. The remaining data are available within the Article, Supplementary Information, or Source Data file.

## Code availability

The developed pipeline and main source R codes that are used in this work are available from Github: https://github.com/XingyiGuo/BC-TFvariants/. The basic computer system requirements include Unix operating system environment, 15–20 GB memory, and R packages (data.table and lme4). It takes about 5 min to run one mixed model for one TF based on our Amazon Web Services (AWS) EC2 machine with m5.24xlarge (96 vCPU and 384 G memory).
