# Supplementary material for: Genetic variations of DNA bindings of FOXA1 and co-factors in breast cancer susceptibility
Source: Nat Commun. 2021 Sep 13;12:5318. doi: 10.1038/s41467-021-25670-9 (PMC8438084; doi:10.1038/s41467-021-25670-9)
Supplement: Supplementary file 1 — Supplementary Information [file 41467_2021_25670_MOESM1_ESM.pdf]

# Genetic variations of DNA bindings of FOXA1 and co-factors in breast cancer susceptibility

Wanqing Wen[1*], Zhishan Chen[1], Jiandong Bao[2], Quan Long[3], Xiao-ou Shu[1], Wei Zheng[1], Xingyi Guo[1,4*]

[1] Division of Epidemiology, Department of Medicine, & [4] Department of Biomedical Informatics, Vanderbilt Epidemiology Center, Vanderbilt-Ingram Cancer Center, Vanderbilt University Medical Center, Nashville TN, 37203, USA.

[2] College of Life Sciences, Fujian Agriculture and Forestry University, Fuzhou 350002, Fujian, China.

[3] Department of Biochemistry and Molecular Biology & Medical Genetics, & Department of Mathematics and Statistics (Adjunct) Member, Alberta Children's Hospital Research Institute, Hotchkiss Brain Institute, O'Brien Institute for Public Health, Calgary, Canada.

* Corresponding author contact information:
Dr. Wanqing Wen
Division of Epidemiology, Department of Medicine
Vanderbilt Epidemiology Center
Vanderbilt University School of Medicine
2525 West End Ave. Suite 600
Nashville, TN 37203
Tel: +1 615-936-0747; Fax: +1 615-332-0502
Email: wanqing.wen@vumc.org;

Dr. Xingyi Guo
Division of Epidemiology, Department of Medicine
Vanderbilt Epidemiology Center, and Vanderbilt-Ingram Cancer Center,
Vanderbilt University School of Medicine
2525 West End Ave. Suite 330
Nashville, TN 37203
Tel: +1 615-936-3471; Fax: +1 615-332-0502
Email: xingyi.guo@vumc.org

**Supplementary Note**

**Supplementary Figure 1: Barplot showing the significance of partitioned heritability for a total of 40 TFs based on LD score regression.**

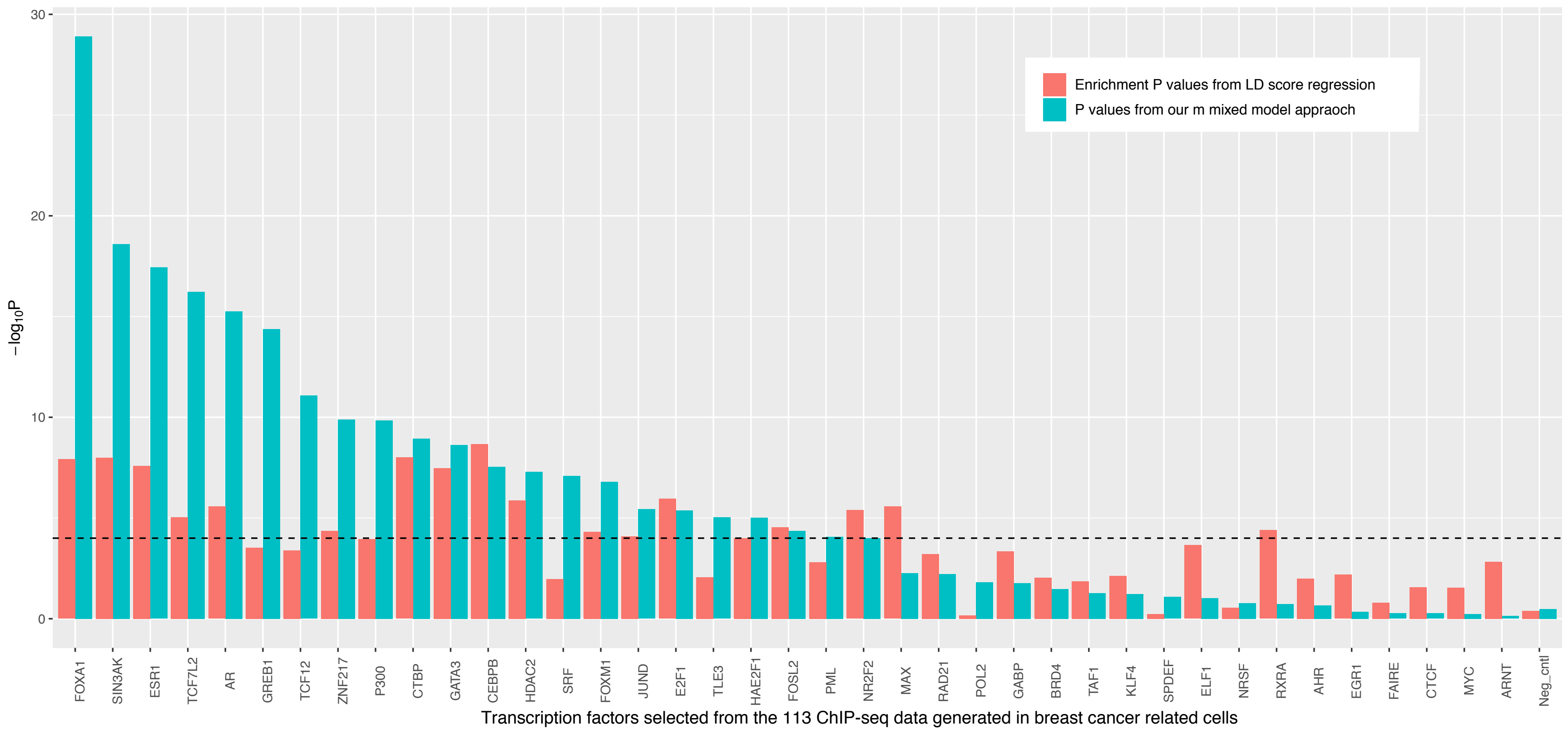

## Supplementary Tables

### Supplementary Table 1: Identified 22 TFs associated with breast cancer risk in BCAC data, at Bonferroni-correction P < 0.05.

| | Association with breast cancer risk | | | | | | Number and percentage of TF-occupied SNPs | | | | | |
|---|---|---|---|---|---|---|---|---|---|---|---|---|
| | Whole genome | | | Deflated genome | | | Whole genome | | 148 GWAS loci† | | GWAS significant SNPs‡ | |
| | N=11337849 | | | N=10139064 | | | N=11337849 | | N=606728 | | N=20887 | |
| **TF** | beta* | se | p | beta* | se | p | N§ | % ‖ | N § | %‖ | N§ | %‖ |
| FOXA1 | 0.1261 | 0.0112 | 1.25E-29 | 0.0155 | 0.0039 | 6.25E-05 | 150264 | 1.33% | 11768 | 1.94% | 688 | 3.29% |
| SIN3AK | 0.0793 | 0.0088 | 2.57E-19 | 0.0068 | 0.0031 | 2.56E-02 | 247692 | 2.18% | 20157 | 3.32% | 844 | 4.04% |
| ESR1 | 0.0835 | 0.0096 | 3.66E-18 | 0.0092 | 0.0033 | 5.64E-03 | 203956 | 1.80% | 16267 | 2.68% | 698 | 3.34% |
| TCF7L2 | 0.1411 | 0.0169 | 5.85E-17 | 0.0345 | 0.0059 | 3.86E-09 | 64782 | 0.57% | 19305 | 3.18% | 254 | 1.22% |
| AR | 0.0791 | 0.0098 | 5.58E-16 | 0.0115 | 0.0034 | 7.14E-04 | 199259 | 1.76% | 15944 | 2.63% | 847 | 4.06% |
| GREB1 | 0.1124 | 0.0143 | 4.18E-15 | 0.0178 | 0.0050 | 3.23E-04 | 90774 | 0.80% | 14903 | 2.46% | 296 | 1.42% |
| TCF12 | 0.1027 | 0.0150 | 8.44E-12 | 0.0129 | 0.0052 | 1.31E-02 | 82188 | 0.72% | 9805 | 1.62% | 321 | 1.54% |
| ZNF217 | 0.1390 | 0.0216 | 1.30E-10 | 0.0236 | 0.0075 | 1.73E-03 | 40559 | 0.36% | 7084 | 1.17% | 156 | 0.75% |
| P300 | 0.1174 | 0.0183 | 1.45E-10 | 0.0088 | 0.0064 | 1.65E-01 | 55087 | 0.49% | 14341 | 2.36% | 246 | 1.18% |
| CTBP | 0.0694 | 0.0114 | 1.16E-09 | 0.0115 | 0.0040 | 3.55E-03 | 144834 | 1.28% | 11630 | 1.92% | 488 | 2.34% |
| GATA3 | 0.0526 | 0.0088 | 2.33E-09 | 0.0085 | 0.0030 | 5.08E-03 | 243784 | 2.15% | 18958 | 3.12% | 803 | 3.84% |
| CEBPB | 0.0540 | 0.0097 | 2.86E-08 | 0.0076 | 0.0034 | 2.46E-02 | 197109 | 1.74% | 15744 | 2.59% | 633 | 3.03% |
| HDAC2 | 0.0552 | 0.0101 | 5.18E-08 | 0.0083 | 0.0035 | 1.79E-02 | 184029 | 1.62% | 15826 | 2.61% | 615 | 2.94% |
| SRF | 0.0700 | 0.0130 | 7.98E-08 | 0.0072 | 0.0045 | 1.12E-01 | 109279 | 0.96% | 11215 | 1.85% | 318 | 1.52% |
| FOXM1 | 0.0854 | 0.0163 | 1.57E-07 | 0.0146 | 0.0057 | 9.97E-03 | 69833 | 0.62% | 8725 | 1.44% | 257 | 1.23% |
| JUND | 0.0633 | 0.0137 | 3.67E-06 | 0.0138 | 0.0047 | 3.55E-03 | 100229 | 0.88% | 11836 | 1.95% | 299 | 1.43% |
| E2F1 | 0.0337 | 0.0073 | 4.13E-06 | -0.0036 | 0.0025 | 1.58E-01 | 375051 | 3.31% | 28249 | 4.66% | 875 | 4.19% |
| TLE3 | 0.0484 | 0.0109 | 9.41E-06 | 0.0075 | 0.0038 | 4.77E-02 | 157002 | 1.38% | 11238 | 1.85% | 344 | 1.65% |
| HAE2F1 | 0.0402 | 0.0091 | 9.58E-06 | 0.0027 | 0.0031 | 3.85E-01 | 234249 | 2.07% | 18309 | 3.02% | 674 | 3.23% |
| FOSL2 | 0.0539 | 0.0132 | 4.31E-05 | -0.0001 | 0.0046 | 9.83E-01 | 106473 | 0.94% | 9392 | 1.55% | 371 | 1.78% |
| PML | 0.0714 | 0.0181 | 8.36E-05 | 0.0145 | 0.0063 | 2.14E-02 | 56420 | 0.50% | 20590 | 3.39% | 155 | 0.74% |
| NR2F2 | 0.0220 | 0.0057 | 1.00E-04 | 0.0052 | 0.0020 | 7.47E-03 | 749498 | 6.61% | 62141 | 10.24% | 1624 | 7.78% |

* The mean difference of the Chi-square values between SNPs with and without the TF occupancy
† A toal of 148 loci were defined in the BCAC data, which include GWAS-identified SNPs and the flanking regions (+/-500Kb). These 148 loci include 606,728 SNPs.
‡ A total of 20,887 SNPs were GWAS-significant with the reported p-value < 5E-8 in the BCAC data.
§ The number of SNPs occupied by the TF
‖ The percentage of the number of SNPs occupied by the TF over the total number of SNPs

**Supplementary Table 2: Genetic variations of TF-DNA binding of TFs associated with breast cancer risk in BCAC data, stratified by motifs (i.e. host, non-host and no motif).**

| | Number of SNPs occupied by TF | | | Host motif | | | Non-host motif | | | No motif | | |
|---|---|---|---|---|---|---|---|---|---|---|---|---|
| TF | Host | Non-host | No motif | beta* | se | p | beta* | se | p | beta* | se | p |
| AR | 46608 | 47107 | 105544 | 0.0882 | 0.0198 | 8.08E-06 | 0.0755 | 0.0197 | 1.25E-04 | 0.0766 | 0.0133 | 8.48E-09 |
| CEBPB | 79736 | 53038 | 64335 | 0.0400 | 0.0151 | 8.11E-03 | 0.0759 | 0.0185 | 4.20E-05 | 0.0535 | 0.0169 | 1.55E-03 |
| E2F1 | 9382 | 73548 | 292121 | -0.0546 | 0.0439 | 2.14E-01 | 0.0944 | 0.0158 | 2.41E-09 | 0.0211 | 0.0082 | 1.01E-02 |
| ESR1 | 10416 | 107877 | 85663 | 0.1805 | 0.0416 | 1.47E-05 | 0.0889 | 0.0131 | 1.01E-11 | 0.0648 | 0.0147 | 1.00E-05 |
| FOSL2 | 25490 | 43955 | 37028 | -0.0135 | 0.0266 | 6.12E-01 | 0.0896 | 0.0204 | 1.09E-05 | 0.0584 | 0.0222 | 8.59E-03 |
| FOXA1 | 50154 | 42988 | 57122 | 0.1368 | 0.0190 | 6.77E-13 | 0.1664 | 0.0206 | 6.47E-16 | 0.0861 | 0.0179 | 1.57E-06 |
| FOXM1 | 4506 | 31192 | 34135 | 0.3908 | 0.0633 | 6.66E-10 | 0.0689 | 0.0242 | 4.40E-03 | 0.0598 | 0.0232 | 9.87E-03 |
| GATA3 | 65373 | 90060 | 88351 | 0.0547 | 0.0167 | 1.05E-03 | 0.0376 | 0.0143 | 8.38E-03 | 0.0663 | 0.0145 | 4.48E-06 |
| JUND | 354 | 44417 | 55458 | -0.2750 | 0.2257 | 2.23E-01 | 0.0561 | 0.0203 | 5.70E-03 | 0.0713 | 0.0183 | 9.60E-05 |
| NR2F2 | 104317 | 112983 | 532198 | 0.0061 | 0.0133 | 6.47E-01 | 0.0543 | 0.0128 | 2.30E-05 | 0.0178 | 0.0066 | 7.00E-03 |
| SRF | 4416 | 53272 | 51591 | 0.0247 | 0.0639 | 6.99E-01 | 0.0873 | 0.0185 | 2.43E-06 | 0.0560 | 0.0189 | 3.00E-03 |
| TCF12 | 6215 | 39713 | 36260 | 0.0897 | 0.0539 | 9.63E-02 | 0.1164 | 0.0214 | 5.71E-08 | 0.0898 | 0.0225 | 6.53E-05 |
| TCF7L2 | 4677 | 30847 | 29258 | 0.1895 | 0.0621 | 2.29E-03 | 0.1674 | 0.0243 | 5.54E-12 | 0.1056 | 0.0249 | 2.33E-05 |
| | | | | | | | | | | | | |
| CTBP | | 93687 | 51147 | | | | 0.0735 | 0.0140 | 1.56E-07 | 0.0616 | 0.0192 | 1.30E-03 |
| GREB1 | | 48625 | 42149 | | | | 0.1574 | 0.0194 | 4.84E-16 | 0.0601 | 0.0209 | 4.04E-03 |
| HAE2F1 | | 65818 | 168431 | | | | 0.0838 | 0.0167 | 5.37E-07 | 0.0231 | 0.0106 | 2.94E-02 |
| HDAC2 | | 83322 | 100707 | | | | 0.0736 | 0.0149 | 7.24E-07 | 0.0399 | 0.0136 | 3.32E-03 |
| P300 | | 31757 | 23330 | | | | 0.1441 | 0.0240 | 1.86E-09 | 0.0810 | 0.0280 | 3.82E-03 |
| PML | | 21807 | 34613 | | | | 0.0499 | 0.0289 | 8.47E-02 | 0.0849 | 0.0231 | 2.28E-04 |
| SIN3AK | | 108795 | 138897 | | | | 0.0839 | 0.0130 | 1.26E-10 | 0.0756 | 0.0116 | 8.28E-11 |
| TLE3 | | 39867 | 117135 | | | | 0.1195 | 0.0214 | 2.29E-08 | 0.0241 | 0.0126 | 5.61E-02 |
| ZNF217 | | 24635 | 15924 | | | | 0.1912 | 0.0272 | 1.99E-12 | 0.0510 | 0.0352 | 1.47E-01 |

* The mean difference of the Chi-square values between SNPs with and without the TF occupancy

**Supplementary Table 3: Association of co-occupancy of three TFs with breast cancer risk in BCAC data, stratified by FOXA1.**

| TF | | | Number of SNPs occupied by TF | | | Both 2nd and 3rd TF | | | Only second TF | | | Only Third TF | | | |
|---|---|---|---|---|---|---|---|---|---|---|---|---|---|---|---|
| First TF | Second TF | Third TF | Both 2nd/3rd TF | The 2nd TF only | The 3rd TF only | beta* | se | p | beta* | se | p | beta* | se | p | P for interaction with FOXA1 |
| FOXA1=0 | ESR1 | E2F1 | 25401 | 117378 | 329246 | 0.0654 | 0.0269 | 1.50E-01 | 0.0037 | 0.0126 | 7.70E-01 | 0.0078 | 0.0078 | 3.14E-01 | |
| FOXA1=1 | ESR1 | E2F1 | 13612 | 47565 | 6792 | 0.4665 | 0.0395 | 3.86E-32 | 0.1632 | 0.0245 | 2.85E-11 | 0.3194 | 0.0538 | 2.87E-09 | 1.80E-18 |
| FOXA1=0 | ESR1 | TCF12V | 20400 | 122379 | 41303 | 0.0930 | 0.0299 | 1.88E-03 | 0.0008 | 0.0123 | 9.47E-01 | -0.0146 | 0.0211 | 4.88E-01 | |
| FOXA1=1 | ESR1 | TCF12V | 18159 | 43018 | 2326 | 0.3244 | 0.0348 | 1.14E-20 | 0.1667 | 0.0251 | 3.18E-11 | 0.2921 | 0.0894 | 1.08E-03 | 6.85E-13 |
| FOXA1=0 | TCF12V | TLE3 | 8625 | 53078 | 126618 | 0.0970 | 0.0459 | 3.47E-02 | 0.0086 | 0.0186 | 6.44E-01 | 0.0074 | 0.0122 | 5.43E-01 | |
| FOXA1=1 | TCF12V | TLE3 | 9806 | 10679 | 11953 | 0.3777 | 0.0448 | 3.37E-17 | 0.1679 | 0.0431 | 9.66E-05 | 0.0346 | 0.0409 | 3.97E-01 | 1.93E-06 |
| FOXA1=0 | SIN3AK | TLE3 | 19043 | 179925 | 116200 | 0.0818 | 0.0309 | 8.24E-03 | 0.0280 | 0.0103 | 6.45E-03 | 0.0025 | 0.0127 | 8.44E-01 | |
| FOXA1=1 | SIN3AK | TLE3 | 16813 | 31911 | 4946 | 0.2879 | 0.0356 | 6.37E-16 | 0.1694 | 0.0275 | 7.46E-10 | -0.0211 | 0.0621 | 7.35E-01 | 2.30E-08 |

* The mean difference of the Chi-square values between SNPs with and without the TF occupancy

**Supplementary Table 4: Association of number of occupied TFs with breast cancer risk in BCAC data.**

| TF score | Number of SNPs occupied by TFs | beta* | se | p |
|---|---|---|---|---|
| ***All 22 TFs combined*** | | | | |
| 0 TF | 9538718 | Reference | | |
| 1-2 TFs | 1450531 | 0.0145 | 0.0040 | 3.15E-04 |
| 3-5 TFs | 196996 | 0.0468 | 0.0098 | 1.87E-06 |
| 4-10 TFs | 104834 | 0.1129 | 0.0133 | 2.61E-17 |
| 11-22 TFs | 46770 | 0.1427 | 0.0199 | 7.02E-13 |
| | | | | |
| ***Score of 21 TFs, stratified by FOXA1*** | | | | |
| FOAX1=0 | | | | |
| 0 TF | 9538718 | Reference | | |
| 1-2 TFs | 1380718 | 0.0143 | 0.0041 | 5.03E-04 |
| 3-5 TFs | 163926 | 0.0297 | 0.0107 | 5.73E-03 |
| 4-10 TFs | 78869 | 0.0450 | 0.0153 | 3.35E-03 |
| 11-21 TFs | 25254 | 0.0066 | 0.0269 | 8.07E-01 |
| FOXA1=1 | | | | |
| 0 TF | 45441 | Reference | | |
| 1-2 TFs | 38969 | 0.0101 | 0.0641 | 8.75E-01 |
| 3-5 TFs | 25128 | 0.2334 | 0.0789 | 3.10E-03 |
| 4-10 TFs | 22675 | 0.2201 | 0.0790 | 5.30E-03 |
| 11-21 TFs | 18051 | 0.3165 | 0.0873 | 2.90E-04 |
| | P for interaction with FOXA1 = 1.23E-18 | | | |

* The mean difference of the Chi-square values between SNPs with and without the TF occupancy
