# Supplementary material for: Genetic variations of DNA bindings of FOXA1 and co-factors in breast cancer susceptibility
Source: Nat Commun. 2021 Sep 13;12:5318. doi: 10.1038/s41467-021-25670-9 (PMC8438084; doi:10.1038/s41467-021-25670-9)
Supplement: Supplementary file 2 — Description of Additional Supplementary Files [file 41467_2021_25670_MOESM2_ESM.pdf]

Description of Additional Supplementary Files

File Name: Supplementary Data 1
Description: A total of 113 ChIP-seq data generated in breast cancer cell lines collected from ENCODE and previous literature.

File Name: Supplementary Data 2
Description: Association of co-occupancy of two TFs with breast cancer risk in BCAC data.

File Name: Supplementary Data 3
Description: : Association of chromatin states with breast cancer risk and interaction with TF score in BCAC data.

File Name: Supplementary Data 4
Description: Breast cancer susceptibility genes identified by TWAS analysis using genetic variants occupied by risk-associated TFs.

File Name: Supplementary Data 5
Description: Susceptibility genes supported by functional genomic data analysis.
