# Supplementary material for: Genetic variations of DNA bindings of FOXA1 and co-factors in breast cancer susceptibility
Source: Nat Commun. 2021 Sep 13;12:5318. doi: 10.1038/s41467-021-25670-9 (PMC8438084; doi:10.1038/s41467-021-25670-9)
Supplement: Supplementary file 4 — Reporting Summary [file 41467_2021_25670_MOESM4_ESM.pdf]

nature research

Corresponding author(s): Xingyi Guo and Wanqing Wen from Vanderbilt University Medical Center

Last updated by author(s): Aug-10-2021

Reporting Summary

Nature Research wishes to improve the reproducibility of the work that we publish. This form provides structure for consistency and transparency in reporting. For further information on Nature Research policies, see our Editorial Policies and the Editorial Policy Checklist.

Statistics

For all statistical analyses, confirm that the following items are present in the figure legend, table legend, main text, or Methods section.

n/a	Confirmed	
☐	☒	The exact sample size (*n*) for each experimental group/condition, given as a discrete number and unit of measurement
☒	☐	A statement on whether measurements were taken from distinct samples or whether the same sample was measured repeatedly
☐	☒	The statistical test(s) used AND whether they are one- or two-sided<br>*Only common tests should be described solely by name; describe more complex techniques in the Methods section.*
☐	☒	A description of all covariates tested
☐	☒	A description of any assumptions or corrections, such as tests of normality and adjustment for multiple comparisons
☐	☒	A full description of the statistical parameters including central tendency (e.g. means) or other basic estimates (e.g. regression coefficient) AND variation (e.g. standard deviation) or associated estimates of uncertainty (e.g. confidence intervals)
☐	☒	For null hypothesis testing, the test statistic (e.g. *F*, *t*, *r*) with confidence intervals, effect sizes, degrees of freedom and *P* value noted<br>*Give P values as exact values whenever suitable.*
☒	☐	For Bayesian analysis, information on the choice of priors and Markov chain Monte Carlo settings
☒	☐	For hierarchical and complex designs, identification of the appropriate level for tests and full reporting of outcomes
☒	☐	Estimates of effect sizes (e.g. Cohen's *d*, Pearson's *r*), indicating how they were calculated

Our web collection on statistics for biologists contains articles on many of the points above.

Software and code

Policy information about availability of computer code

Data collection	No software was used for data collection.
Data analysis	BWA mem program (version 0.7.9a) was used for ChIP-seq raw reads mapping to human genome (hg 19).<br><br>MACS tool (version 1.4) was used for peak binding calling of transcription factors.<br><br>ChIPseeker (version 1.28) was used for the annotation of global binding occupancy for each TF in the human genome.<br><br>HOMER (version 2) was used for motif discovery and enrichment analysis.<br><br>We used continuous Chi-squared value for each genetic variant reported in the BCAC GWAS summary data to measure its association with breast cancer risk. We then used the generalized mixed models under the Gaussian distribution to estimate the associations between the Chi-squared values (Y) and TF binding status of genetic variants located in binding sites of each TF, given LD blocks of genetic variants to handle the dependence between genetic variants. We defined the LD blocks using non-overlapping segments of 100kb (a similar result with 500kb). We conducted similar analyses for the binary GWAS p-values cut at a certain threshold (e.g., $P < 5 \times 10$-8) using the generalized mixed models under the binomial distribution to estimate their associations with TF binding status of genetic variants .<br><br>The analysis R codes and detailed description of the data analysis are available via https://github.com/XingyiGuo/BC-TFvariants.

For manuscripts utilizing custom algorithms or software that are central to the research but not yet described in published literature, software must be made available to editors and reviewers. We strongly encourage code deposition in a community repository (e.g. GitHub). See the Nature Research guidelines for submitting code & software for further information.

Data

Policy information about availability of data

All manuscripts must include a data availability statement. This statement should provide the following information, where applicable:

- Accession codes, unique identifiers, or web links for publicly available datasets
- A list of figures that have associated raw data
- A description of any restrictions on data availability

All the analysis data can be publicaly accessed through the parent studies, databases or URLs.

Main analysis data: Summary statistics of GWAS data for breast cancer were downloaded from the Breast Cancer Association Consortium (BCAC). ChIP-seq data in breast cancer cell lines were collected from the Encyclopedia of DNA Elements (ENCODE) and the Cistrome database (http://cistrome.org/).

Gene-expression prediction model building: Gene expression and genotype data in breast cancer were collected from the Genotype-Tissue Expression (GTEx), The Cancer Genome Atlas (TCGA) and the Molecular Taxonomy of Breast Cancer International Consortium (METABRIC).

Functional annotation: Chromatin states annotation in HMEC (Roadmap E028 cell) and myoepitheilal primary cells (Roadmap E027 cell) were downloaded from Roadmap Project.

CRISPR-Cas9 essentiality screens: To investigate the effect of an individual gene on essentiality for proliferation and survival of cancer cells, we collected two comprehensive datasets including “sample_info.csv” and “Achilles_gene_effect.csv” from the DepMap portal (https://depmap.org/portal/).

Knockdown experiment data: Gene expression data from FOXA1 knockdown experiments for FOXA1 in breast cancer MCF7 cells were downloaded from NCBI using accession number GSE25315. Gene expression data from small hairpin (shRNA) plasmid transfection to silence ESR1 in MCF7 and over-expression for GATA3 in MDA-MB-231 cell lines were downloaded from NCBI using accession numbers GSE27473 and GSE24249, respectively.

Field-specific reporting

Please select the one below that is the best fit for your research. If you are not sure, read the appropriate sections before making your selection.

- [x] Life sciences
- [ ] Behavioural & social sciences
- [ ] Ecological, evolutionary & environmental sciences

For a reference copy of the document with all sections, see nature.com/documents/nr-reporting-summary-flat.pdf

Life sciences study design

All studies must disclose on these points even when the disclosure is negative.

Sample size	This study used publicly available datasets, including the GWAS data from the Breast Cancer Association Consortium (n=122,977 cases and n=105,974 controls in total), 113 Chip-seq datasets, gene expression datasets from GTEx (n=85), TCGA (n=536), and METABRIC (n=1,891). We have adequate statistical power to identified breast cancer risk associated transcription factors and susceptibility genes (see Result section).
Data exclusions	We analyze the summary statistics of GWAS data, thus no data exclusions were performed.
Replication	This study was to develop an analytic approach using publicly available data. We compared our approach with existing approaches, such as LD score regression. This study did not produce data from individual study participants, therefore, it is not applicable to conduct replication study.
Randomization	Randomization design is not applicable, because this study was to develop an analytic approach using publicly available data.
Blinding	Blinding is not applicable, because this study was to develop an analytic approach using publicly available data.

Reporting for specific materials, systems and methods

We require information from authors about some types of materials, experimental systems and methods used in many studies. Here, indicate whether each material, system or method listed is relevant to your study. If you are not sure if a list item applies to your research, read the appropriate section before selecting a response.

Materials & experimental systems

n/a	Involved in the study
☒	☐ Antibodies
☒	☐ Eukaryotic cell lines
☒	☐ Palaeontology and archaeology
☒	☐ Animals and other organisms
☒	☐ Human research participants
☒	☐ Clinical data
☒	☐ Dual use research of concern

Methods

n/a	Involved in the study
☐	☒ ChIP-seq
☒	☐ Flow cytometry
☒	☐ MRI-based neuroimaging

ChIP-seq

Data deposition

☒ Confirm that both raw and final processed data have been deposited in a public database such as GEO.

☐ Confirm that you have deposited or provided access to graph files (e.g. BED files) for the called peaks.

Data access links<br>*May remain private before publication.*	ChIP-seq data in breast cancer cell lines were collected from the ENCODE and the Cistrome database (http://cistrome.org/). We have provided the detailed information for each dataset in our supplementary table 1.
Files in database submission	Not applicable.
Genome browser session<br>(e.g. UCSC)	Not applicable.

Methodology

Replicates	Not applicable.
Sequencing depth	Not applicable.
Antibodies	Not applicable.
Peak calling parameters	macs14 -t TF-ChIP-Seq.bam -c TF-input.bam (if available) -f BAM -n TF -g hs -p 1e-3 -w; Binding peaks were identified using a stringent criterion at a score > 30.
Data quality	We have manually evaluated the data quality based on the data description from parent studies.
Software	MACS tool (version 1.4) (PMID: 18798982).
